# A blended professional learning intervention for early childhood educators to target the promotion of physical activity and healthy eating: the HOPPEL cluster randomized stepped-wedge trial

**DOI:** 10.1186/s12889-022-13542-w

**Published:** 2022-07-15

**Authors:** Peden ME, Eady MJ, Okely AD, Patterson K, Batterham M, Jones RA

**Affiliations:** 1 Early Start, School of Education, Faculty of the Arts, Social Sciences and Humanities, University of Wollongong, Wollongong, NSW 2522 Australia; 2 School of Education, Faculty of the Arts, Social Sciences and Humanities, University of Wollongong, Wollongong, NSW 2522 Australia; 3 School of Health and Society, Faculty of the Arts, Social Science and Humanities, University of Wollongong, Wollongong, NSW 2522 Australia; 4 Faculty of Education, University of Tasmania, Tasmania, 7005 Australia; 5 School of Mathematics and Applied Statistics, University of Wollongong, Wollongong, NSW 2522 Australia

**Keywords:** Physical activity, Healthy eating, Blended professional learning, Early childhood education and care, Children, Intervention

## Abstract

**Background:**

Childcare centres are important environments for promoting physical activity and healthy eating. Blended approaches to professional learning may help overcome existing challenges for educators in promoting these behaviours. This study aimed to test the effect of a blended professional learning program on healthy eating and physical activity in childcare.

**Methods:**

Cluster randomized stepped-wedge trial in 15 childcare centres in Tasmania, Australia. Children aged 2-5y who attended at least two days per week were eligible to participate. Random assignment occurred at the centre level. Centre names were drawn out of a hat and then subsequently allocated to one of the three steps. The intervention comprised a 12-week blended professional learning program for educators. The main outcome was centre-level physical activity and healthy eating, assessed using the Environment and Policy Assessment Observation System (EPAO). All data collectors were blinded to step allocation. Analyses were according to intention to treat. The trial was registered with the Australian New Zealand Clinical Trial Registry (ACTRN12618000346279, date registered: 07/03/2018).

**Results:**

Centres were recruited between January 2016 and February 2016. All centres were retained for the duration of the study. A total of 313 children were recruited with 291 analysed at the completion of the study (93%). The difference between groups for the EPAO total score was significant at the end of the maintenance period (adjusted difference = 14.63, 95% CI [1.33, 27.92], *p* = 0.03). Significant differences were found for the percentage of time children spent in light-intensity physical activity at the end of the intervention (adjusted difference = 0.01, 95% CI [0.00,0.01], *p* = 0.02) and maintenance periods (adjusted difference = 0.01, 95% CI [0.00,0.02], *p* = 0.04). To the best of the authors knowledge, there were no adverse events.

**Conclusion:**

This intervention achieved a sustained improvement in physical activity and healthy eating in childcare centres. Further, it can be easily integrated into existing service provision, especially among centres with limited access to professional learning.

**Trial registration:**

The study was registered with the Australian and New Zealand Clinical Trials Registry (ACTRN12618000346279, date registered: 07/03/2018).

## Background

Early childhood education and care (ECEC) settings are important environments for targeting young children’s physical activity and healthy eating [1]. The U.S. National Academy of Medicine recommend that children should be active for at least 15 min per hour while in ECEC (with limited sitting or standing time). ECEC settings provide a variety of healthy foods and age-appropriate portion sizes and promote the consumption of water [2]. Only 50% of children currently meet these recommendations for physical activity and a high proportion of children do not meet dietary guidelines [3, 4]. As such, innovative and sustainable ECEC-focused interventions that promote physical activity and healthy eating are needed.

A range of intervention approaches (e.g., length, resources, type of facilitator etc.) have been used to promote physical activity and healthy eating in ECEC centres. Irrespective of the approach, what is important and well recognised is the role of the educator. Most interventions that promote physical activity and healthy eating usually involve some type of professional learning for educators [5]. Professional learning varies considerably in duration and length; from a few hours to multiple full-day sessions [6]. Despite the variations in length and duration, most professional learning for the ECEC sector is delivered using traditional one-off, face-to-face workshops involving one educator from each centre [5]. However, this type of professional learning is associated with several limitations (e.g., scheduling, cost, knowledge transfer, reach), thus alternative professional learning models are needed for the ECEC sector.

Blended professional learning models (i.e., professional learning inclusive of a face-to-face component and an online component) have successfully changed educator behaviours in the field of education [7, 8]. These models provide educators with convenient access and greater flexibility to access learning materials and increased ongoing opportunities to reflect upon professional learning content and share knowledge and resources in an online communal space [7]. Of note is the ability of these models to reach educators in rural and remote areas addressing the opportunities they have available for professional learning. Furthermore, educators can participate in a virtual community of practice, whereby opportunities of collaboration, enhanced learning and strong professional relationship building, and mentoring are established and maintained in a virtual community. Blended professional learning models to date, have not been assessed as an approach in the promotion of healthy eating and physical activity in ECEC settings. The aim of this study was to evaluate the efficacy of a ‘blended’ professional learning program for early childhood educators on the physical activity and healthy eating environments and policies and on the physical activity levels of children in ECEC services.

## Methods

### Study design

**Fig. 1:**
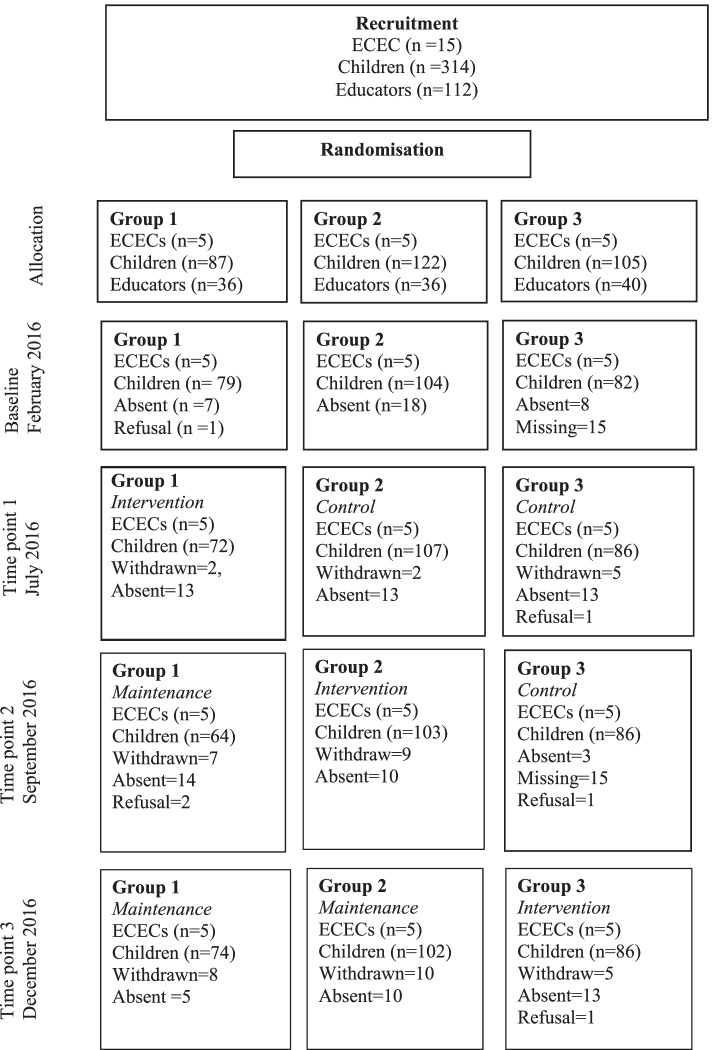
Trial profile for the HOPPEL professional learning program (Stepped wedge modified CONSORT diagram)

A stepped-wedge clustered randomized controlled trial (SW-CRCT) design was used. ECEC centres were randomly allocated to three groups: five ECEC centres formed a group. The groups were then subsequently randomly allocated to steps. The group allocated to step One participated in the blended professional learning intervention first, followed by those in allocated to step Two, followed by those allocated to step Three. The study was conducted in ECEC centres in Tasmania, Australia. The study followed the Consolidated Standards of Reporting Trials (CONSORT) Statement with extension and the flow of the study is depicted in Fig. 1 [9].

### Participants

ECEC educators and children were recruited from ECEC centres from one overarching administrating organization. Centres that catered for children aged 2–5 years within the targeted organization were eligible to participate in the study. Excluded from the study were (1) children less than 2 years old, (2) children aged 2–5 years enrolled for less than two days per week, (3) special population groups (children with diagnosed physical disabilities). Educators in each of the participating centres assisted the primary researcher (MP) in inviting families and children to participate in the study. All educators were employed at centres in outer regional or remote locations by one organization. Greater than 60% of educators at each center had a formal ECEC qualification and had been employed in the organization for at least three years. Most educators (84%) had not previously participated in professional development relating to physical activity nor participated in a blended professional development [10]. All participants had access to the internet, although the internet connectivity varied between participants depending on their geographical location and subsequent internet activity.

### Randomization and masking

Randomization was a two-step process, as per the stepped wedge design. Random assignment occurred firstly at the centre level. Centres names were drawn out of a hat by a researcher from the research group of the authors, who was not involved in the study in any other capacity. Centres were randomized to one of three groups (i.e., each group comprised five centres). The groups were then further randomized into steps. Centres randomized to Step One participated in the intervention first, followed by the group randomized to Step Two and then finally Step Three. All data collectors were blind to group allocation. The educators were not blind to group allocation.

### Intervention

The intervention was facilitated three consecutive times, once for each group (five ECEC centres). The intervention is a 12-week blended professional learning program for ECEC educators. The program, known as HOPPEL (Healthy Online Professional Program for Early Learners), aligned with the physical domain of child development, and focused on physical activity and healthy eating for children aged 2–5 years. Despite the physical domain being a fundamental component in several ECEC curricula, it is often overlooked within ECEC practices [11, 12]. Previously, educators indicated they had not received professional learning in this area, leading to limited confidence and competence levels in delivering this domain in practice [13].

HOPPEL focused on a number of physical activity components, including structured and unstructured physical activity learning experiences, inside and outside physical activity, activity ‘power’ breaks with the aim of breaking up sedentary time and, designing holistic learning environments that promote physical activity. In relation to healthy eating, the content covered: strategies to increase water intake in the outdoor and indoor environments, suggestions on how to increase milk and fruit and vegetable consumption and, ideas about promoting healthy eating behaviours across all aspects of the daily routine. Components synonymous with both physical activity and healthy eating behaviours such as policy development and promoting family partnerships were also included.

HOPPEL consisted of a face-to-face six-hour professional learning workshop, followed by 12 weeks of online professional learning. The online elements comprised of: asynchronous weekly blogs posted by the expert/lead researcher; asynchronous forums that acted as a medium for educators and the lead researcher to communicate and share ideas and resources on the content areas; and three scheduled synchronous online sessions offered via an online learning platform (Adobe Connect, version 9). Each synchronous session lasted approximately one hour and were conducted in the evening with educators logging on at home or during nightly staff meetings. Educators were mentored during the online activities, weekly challenges, and professional discussions throughout the program. The online activities varied and included: presentations from the researchers detailing current research, breakout rooms, and whiteboarding to enable collective brainstorming and interaction between educators, and chat functions, whereby questions were answered in real time. Educators were also encouraged to write short blogs that demonstrated reflection on practices relating to promoting physical activity or encouraging healthy eating for young children. Some of the weekly challenges included: (1) promoting physical activity and healthy eating messages to families through displays; (2) increasing child’s agency at mealtimes by promoting and encouraging self-serving of food (for children older than 2-years); (3) designing physical activity experience using recycled materials; (4) modifying traditional sedentary experiences into more active experiences and; (5) increasing water intake by introducing flavoured water using fruit. The activities and challenges were complimented by professional discussions on topics such as: (1) what are power breaks and fundamental movement skills?; (2) increasing physical activity and decreasing sedentary behaviours across every day indoor and outdoor experiences and; (3) the importance of role modelling and positive interaction and engagement. During the control period all centres continued with usual practice and during the maintenance period, ongoing access to the asynchronous component and resources posted during the intervention period were available.

HOPPEL aligned with two theoretical frameworks: Guskey’s model of teacher change [14] and the Community of Practice [14]. Guskey’s model is based on meaningful, intentional, ongoing and structured professional learning focused on increasing knowledge, skills, attitudes and levels of self-efficacy [14]. This was operationalized by providing a highly innovative and engaging blended professional learning program in physical activity and healthy eating behaviours, an area which is under-represented in practice within the ECEC setting. This model recognizes the importance of the flow-on effects of teacher change on child outcomes. As such, HOPPEL also focused on the impact of the educator’s professional learning on child outcomes. The Community of Practice evolved from Vygotsky’s, sociocultural theory of learning [15]. It emphasizes the importance of social interactions in the learning process and suggests that successful learning is underpinned by the construction of ‘learning communities or networks’, where individuals feel connected to others and are able to discuss and solve problems in a supportive environment. Such communities also aim to provide an environment for individuals to capture and share knowledge, generate new knowledge, and stimulate learning and collaborative processes to help improve practice [16]. Furthermore, the Community of Practice theory encourages members of a community to share common interests and goals around a joint interest to improve skills by working alongside more experienced members [16, 17]. The Community of Practice Theory is based on three fundamental elements (Domain, Community and Practice) and number of associated sub-elements. This was operationalized by encouraging the formation of ‘communities of practice’. In this study three ‘communities of practice’ were developed: each group (comprising five ECEC centres) formed a ‘community of practice’. Educators in each group were given opportunities to share their experiences and resources, develop supportive professional networks and reflect on current pedagogical practices and in turn modify current pedagogical practices through both face-to-face and online professional learning sessions. The intervention was facilitated three times, once for each group, thus the ‘communities of practice’ were individual entities.

Baseline data were collected in all ECECs. In Step 1, Group One participated in the intervention while the other groups maintained usual practice. Data were then collected again in all centres. In Step 2, Group Two participated in the intervention. Group Three continued with usual practice and Group One started the maintenance period (which involved the ECEC centres continuing to implement changes within their centres with reduced support). This process was repeated again in Step Three, with Group One continuing in the maintenance period, Group Two entering the maintenance period and Group Three participating in the intervention. Final data collection was then conducted. As per the stepped-wedge design, the control and maintenance periods varied. At each time point centre- and child-level data were collected.

### Outcomes

The primary outcome was changes in centre-level physical activity and healthy eating practices, which were assessed using the Environmental Policy Assessment and Observation (EPAO) tool [18]. The secondary outcome was changes in children’s physical activity.

The EPAO assesses the physical activity and healthy eating environment and practices of ECECs [18]. It is a valid and reliable observation-based instrument that involves one-day of continual observation. Prior to data collection, all data collectors participated in specific EPAO training with the inter-observer agreement between observers being 78%. Data collectors positioned themselves in non-obtrusive positions within the ECECs and did not disrupt normal routines or activities. Data collectors accessed documents such as policies/procedures pertaining to healthy eating and physical activity, guidelines for celebration foods, fundraising materials, past and present menus, daily program schedules and a copy of the centre layout. Educational materials for parents, curriculum materials and training materials for staff associated with the promotion of healthy eating and physical activity were reviewed. Safety documents pertaining to indoor and outdoor learning environments were checked.

Each of the 16 subscales (8 for physical activity and 8 for healthy eating) were scored according to previous studies [18]. All item responses were converted to a three-point scale (ranging from 0–2). For all 16 subscales, the converted responses were tallied and divided by the number of items present in each subscale. In seven centres, the food was not supplied by the ECEC centre, rather, children supplied their own food. In these instances, the numbers of items tallied were adjusted to standardize scoring across all centres. Adding the individual subscale scores derived a total physical activity score and a total healthy eating score. Adding the total physical activity score and the healthy eating score derived an overall total EPAO score.

Children’s physical activity was assessed using Actigraph GT1M and GT3X + accelerometers. Educators placed the accelerometers on the right hip of consenting children on arrival to the centre each day and then removed it at the end of the day. The epoch length was set to 15-s intervals [19]. Data were considered valid if a child accumulated 180 min on at least one day [20]. Twenty minutes of continuous zeros was considered non-wear time during analysis. The Pate modified cut-points were used to define sedentary behaviour (< 100 counts/min); low light-intensity physical activity (low LPA) (101–800 counts/min); high light-intensity physical activity (801–1679 counts/min); moderate- (1680–3367 counts/min); vigorous- (> 3368 counts/mins); moderate- to vigorous-intensity PA (MVPA) (> 1680) [21]. For this study, only high LPA was used and referred to thereafter as light physical activity (LPA). Total PA was operationalized as time was spent in light, moderate and vigorous intensity physical activity (LMVPA). To the best of the authors’ knowledge, no adverse events which were related directly to the study were reported throughout the implementation period from any educator or children involved in the study.

### Statistical analysis

The sample size for the study was calculated based on the centre-level EPAO outcome for physical activity. Based on changes in the physical activity component of the EPAO of 2.8 units, assuming a SD of 1.15, the estimated number of centres required was 11. As attrition is common in stepped-wedge designs 15 centres were recruited [22]. At the child level the minimum detectable difference based on the proposed design was 4% in total physical activity (LMVPA). All calculations were performed using STATA v14. The effects of the intervention were tested using a multi-level mixed effects linear regression model. The analysis was performed using the mixed syntax and included, group (treatment or control) and steps (time period) as categorical variables and centre as clusters for the centre level variables. An additional level including child ID was included for the child level variables.

### Ethical approval

This study was conducted in accordance with the Declaration of Helsinki and ethics approval for the study was obtained from the Human Research Ethics Committee, University of Wollongong, Australia (HE15/356). Written informed consent was obtained from all of the early childhood education and care educators involved in the study. Parents of children attending the early childhood education and care settings, where the study was conducted, signed a written informed consent from on behalf of their child. Children provided verbal assent. The study protocol is available on the ANZCTR website (ACTRN12618000346279, date registered: 07/03/2018).

## Results

Centres and participants were recruited between January and February 2016. A total of 15 ECEC centres, 104 educators (female = 85%) and 313 children (mean child age = 3.25 years, girls = 46%) were recruited (see Fig. 1). All ECEC centres were retained, and data were collected in all centres at baseline, at the end of the intervention period (12-weeks) and at the end of the maintenance period. Ninety educators and 289 children were retained in the study (79% and 92%, respectively). Twenty-three children left the participating ECEC centres during the study (see Fig. 1). To the best of the authors knowledge, no educator or child left the study for reasons related to the study. Table 1 displays participant (child and educator) characteristics. Most educators were aged between 30–39 years and had diploma level training. Fewer educators were employed on a full-time basis, with the majority of educators employed for the participating organization for 3–5 yrs.

**Table 1:** Baseline characteristics of educators involved in the HOPPEL professional learning program

**Characteristic**	**Number (%)** (total *n* = 104)
**Age**	
Under 25 years	15 (14)
26–29 years	23 (22)
30–39 years	34 (33)
40–49 years	22 (21)
50–59 years	10 (10)
**Highest qualification**	
Certificate	32 (31)
Diploma	47 (45)
Bachelor degree	16 (15)
Other	9 (9)
**Employment status**	
Full-time	37 (36)
Part-time	65 (63)
No Response	2 (1)
**Time employed as educator**	
< 1 years	1 (1)
1–2 years	18 (17)
3–5 years	30 (29)
6–8 years	8 (8)
> 8 years	47 (45)
**Time employed within organisation**	
< 1 years	8 (8)
1–2 years	26 (25)
3–5 years	29 (28)
6–8 years	14 (13)
> 8 years	27 (26)
**Current position**	
Manager	11 (10)
Educational Leader	1 (1)
Teacher (2^nd^ in charge)	3 (3)
Room leader	24 (23)
Educator	65 (63)

Centre-level results are summarised in Table 2. The total EPAO score was not significantly different between the control and intervention groups at post-intervention (adjusted difference = 8.94, 95%CI [-0.22,18.09], *p* = 0.06), but was significant at the end of the maintenance period (adjusted difference = 14.63, 95% CI [1.33, 27.92], *p* = 0.03). For the total physical activity EPAO score, a significant difference was observed between the intervention and control groups at the end of the intervention period (adjusted difference = 5.33, 95% CI [-0.30,10.37], *p* = 0.04), and this difference increased by the end of the maintenance period (adjusted difference = 8.54, 95% CI [1.61,15.48], *p* = 0.02).

**Table 2:** Differences between groups in physical activity and healthy eating outcomes for early childhood education and care settings participating in the HOPPEL professional learning program

	**Post-Intervention Period**				**Post-Maintenance Period**			
	**Control**	**Int**	**Coeff(95%CI)**	***p*-value**	**Control**	**Int**	**Coeff(95%CI)**	***p*-value**
**EPAO-HE**	101.81 ± 4.11	105.41 ± 4.75	3.60 (2.98,10.19)	0.28	100.09 ± 4.73	105.33 ± 4.73	5.24 (-4.65,15.12)	0.30
**EPAO-PA**	109.72 ± 1.56	115.09 ± 2.40	5.33 (0.30,10.37)	**0.04**	106.81 ± 2.50	115.36 ± 2.50	8.54 (1.61,15.48)	**0.02**
**Total EPAO**	211.56 ± 4.47	220.49 ± 5.56	8.94 (-0.22,18.09)	0.06	206.48 ± 5.50	221.10 ± 5.50	14.63 (1.33,27.92)	**0.03**
**SB**	0.62 ± 0.02	0.61 ± 0.02	-0.01 (-0.03, 0.01)	0.20	0.63 ± 0.02	0.60 ± 0.02	-0.02 (-0.05,0.01)	0.11
**LPA**	0.11 ± 0.01	0.12 ± 0.01	0.01 (0.00,0.01)	**0.02**	0.10 ± 0.01	0.12 ± 0.01	0.01 (0.00,0.02)	**0.04**
**MPA**	0.10 ± 0.01	0.10 ± 0.01	0.00 (-0.01,0.01)	0.86	0.10 ± 0.01	0.11 ± 0.01	0.01 (-0.00, 0.02)	0.12
**VPA**	0.03 ± 0.00	0.03 ± 0.00	-0.00 (-0.00,0.00)	0.70	0.03 ± 0.00	0.03 ± 0.00	-0.00 (-0.00,0.01)	0.66
**MVPA**	0.13 ± 0.01	0.13 ± 0.01	0.00 (-0.01,0.01)	0.80	0.13 ± 0.01	0.14 ± 0.01	0.01 (-0.01,0.02)	0.19
**LMVPA**	0.24 ± 0.02	0.25 ± 0.02	0.01 (-0.01,0.03)	0.26	0.23 ± 0.02	0.25 ± 0.02	0.02 (-0.00,0.05)	0.10

The results for child-level physical activity data are also presented in Table 2. A significant difference in percentage of time spent in light physical activity was reported between control and intervention groups at the end of the intervention period (adjusted difference = 0.01, 95% CI [0.00,0.01], *p* = 0.02] and this was maintained at the end of the maintenance period (adjusted difference = 0.01, 95% CI [0.00,0.02], *p* = 0.04).

All of the face-to-face professional learning sessions for each group was facilitated as planned. All face-to-face professional learning sessions ran on time and all content was delivered as planned. Additionally, for all groups, the three online synchronous sessions were facilitated as intended. Additional information pertaining to the number of participants in the sessions and feedback from educators regarding the face-to-face professional learning sessions and the synchronous online sessions has been recently published [10]. To the authors’ knowledge, no participants were hurt during the implementation of the intervention.

## Discussion

The results of this stepped-wedge randomized controlled trial show that HOPPEL, a blended program professional learning program for ECEC, was efficacious in eliciting significant positive changes in centre- and child-level physical activity outcomes. Given the uniqueness of this blended professional learning program in an ECEC setting, the findings of this program are noteworthy. The importance of educators participating in an alternative professional learning model is a promising approach for promoting healthy eating behaviours and physical activity in ECEC settings and warrants further investigation in the future.

Significant effects in total EPAO score, EPAO for physical activity scores and light physical activity were found at the end of the intervention period. The significant changes in physical activity increased at the end of the maintenance period, providing evidence that these changes can be sustained. To the authors’ knowledge, only one study has simultaneously reported changes in both the physical activity and healthy eating EPAO components. Similar to this study, Lyn et al. [23] reported significant changes in the total physical activity EPAO score (*p* < 0.001) at the end of the intervention period (12-months). This study extends these findings by reporting on the total EPAO score and measures effects at the end of the maintenance period. Furthermore, HOPPEL included results on the changes in objectively measured child physical activity to supplement the results from the direct observational tool.

The significant changes in the centre-level and child-level physical activity outcomes can be attributed to the educators’ level of engagement with the HOPPEL professional learning program. This finding is consistent with previous studies reporting the value of ongoing professional learning programs [24, 25]. Data from a recent study which also implemented a professional learning intervention (focusing on a different content area) and measured centre- and child-level outcomes showed that several professional learning sessions were far superior than a one-off professional learning session. The same study showed that involving more educators in the professional learning was also superior than just involving one educator [24]. These principles were similar to those in this study, where a number of ongoing professional learning sessions were offered over a 12-week period and all educators were encouraged to participate in the face-to-face professional learning session, as well as the online component of the professional learning.

In this study, baseline data were made available to all centres at the beginning of the intervention period, enabling the content of the professional learning to be tailored to meet the specific needs of each centre. Given that physical activity and healthy eating behaviours are often unrepresented within the ECEC context, it was important to highlight key areas where centres were performing well, as well as highlight areas for improvement. The synchronous online sessions provided regular opportunities for educators to communicate, share and collaborate with the expert and their colleagues [16]. It was in this environment, that educators could speak freely about their new knowledge and skills. This ongoing collaboration and familiarity with other educators may have encouraged educators to make sustainable changes within their settings.

The physical activity content, which was delivered as part of HOPPEL may have contributed to the changes reported in physical activity. In contrast to other studies [26, 27], a prescribed amount of physical activity was not mandated throughout the intervention, rather the content provided suggestions related to physical activity learning experiences, as well as probing questions for educators to discuss in staff meetings and weekly challenges. Furthermore, the content also focused on the importance of the ECEC environment and the role of the educator in terms of offering physical activity opportunities for children. This approach aligns with the philosophy of educators and perhaps educators felt less threatened by this approach and were more willing to provide enhanced physical activity opportunities for the children. Further exploration of this was beyond the scope of the study, however could be investigated further in future studies.

While many studies have reported on changes in objectively measured physical activity [27–29] at the end of an intervention, fewer studies have reported sustainable significant changes beyond the intervention period (i.e., during a maintenance period). After educators participated in the blended professional learning program for 12 weeks, educators entered the maintenance period whereby they were still able to access the online forum to exchange ideas, however synchronous weekly blogs and asynchronous live chat sessions facilitated by the lead researcher ceased. During this maintenance period, significance changes in physical activity continued, which could be attributed to educators’ willingness to engage in ongoing supportive peer behaviours, educators' ability to independently reflect upon and showcase changes to pedagogical practices on a specified topic, and educators’ increased knowledge and skills which led to enhanced levels of confidence and autonomy in promoting physical activity practices. Thus, the positive changes recorded in the present study are perhaps more significant and meaningful as HOPPEL focused on two areas: physical activity and healthy eating within one intervention.

The absence of significant findings in the healthy eating EPAO score at the end of the intervention as well as the end of the maintenance period could be related to the high number (46%) of centres that were lunch box only centres. A lunch box centre is where parents/carers are asked to provide children’s food (snacks and lunch) whilst attending the centre. The remaining centres provided children with all meals. Therefore, within this study, a true audit could not be completed using the EPAO given the participating centres were all operating under different eating occasions and use of menus.

This study has several strengths. First, it adopted a stepped-wedge design that allowed all groups to act as their controls and allowed for all groups to receive the intervention. This is one of the first studies within the ECEC sector to adopt such a design. The SW-RCT is becoming increasingly more utilised in interventions because of ethical reasons, for example, by all centres receiving the intervention, the control groups were not denied the hypothesized benefits of the intervention [22]. Additionally, the stepped-wedge has an inbuilt maintenance period, allowing data to be collected from centres over a prolonged period of time (i.e., in this study over a 12-month period). A second strength is the reporting of both centre- and child-level data using validated instruments. Third, this study was underpinned by strong foundational frameworks that aimed to increase the knowledge and skills of educators via a blended professional learning program, whilst accounting for the impact on child learning outcomes. Fourth, the study recruitment and retention rates were high, with all centres remaining in the study and more than 90% of children being retained, suggesting high feasibility of such an approach. Finally, this study employed a novel and alternative form of professional learning to elicit changes in children’s physical activity and healthy eating behaviours that has not been previously reported.

There were several limitations in this study. Although the SW-CRCT design offers a number of advantages over traditional intervention designs, it involves a number of additional data collection points, thus data collection is more costly and time consuming [22]. Second, in this study the collection of data for time points 2 and 3 coincided with school holidays, resulting in increased absenteeism of children which may have potentially impacted the changes in child-level data reported. A large portion (46%) of the centres did not provide the food for the children throughout the day (i.e., the children brought their food from home), a practice that is not uncommon in some ECEC centres, in Australia. Therefore, this may have impacted the centre-level healthy eating component of the EPAO. Third, we did not collect data pertaining to internet connectivity. All educators had access to the internet, however connectivity varied which on occasions was a limiting factor for educator’s engagement. It may have been plausible, although expensive, to upgrade the internet connectivity for some centers, however to ensure transferability and up-scaling of this intervention it was important to facilitate the intervention in real world conditions.

## Conclusion

To our knowledge, this is the first study to evaluate the efficacy of a blended professional learning program for ECEC educators, targeting both physical activity and healthy eating among 2–5-year old’s and using a stepped-wedge design. In contrast to many other studies within the ECEC sector, significant results were reported for the physical activity outcomes at the end of the intervention period, which were increased at the end of the maintenance period. The ECEC environment is a critical setting for the promotion of physical activity and healthy eating behaviours [1] and thus interventions need to be effective yet innovative in their approach. The HOPPEL program addresses both of these criteria and has the potential to be used widely across all geographical and socioeconomic ECEC settings. Equipping educators with the knowledge and skills to promote physical activity and healthy eating is paramount for children’s health and wellbeing.

## Data Availability

Data supporting the results reported in this article are stored at the University of Wollongong. These data are available upon request by contacting the first author.

## Abbreviations

CI: Confidence interval
ECEC: Early childhood education and care
EPAO: Environment and Policy Assessment Observation System
High LPA: High light physical activity
HOPPEL: Healthy Online Professional Program for Early Learners
Low LPA: Low light-intensity physical activity
LMVPA: Light, medium and vigorous physical activity (total physical activity)
MVPA: Moderate-to-vigorous-intensity physical activity
SD: Standard deviations
SW-CRCT: Stepped wedge clustered randomized controlled trial
